# Overexpression of MicroRNA-145 Ameliorates Astrocyte Injury by Targeting Aquaporin 4 in Cerebral Ischemic Stroke

**DOI:** 10.1155/2017/9530951

**Published:** 2017-09-13

**Authors:** Lifang Zheng, Wei Cheng, Xijia Wang, Zhigang Yang, Xiangling Zhou, Chunlian Pan

**Affiliations:** Department of Neurology, The Puren Hospital, Wuhan University of Science and Technology, Wuhan 430081, China

## Abstract

Cerebral ischemic stroke, which affects the global population, is a major disease with high incidence, mortality, and disability. Accumulating evidence has indicated that abnormal microRNA (miRNA) expression plays essential roles in the pathologies of ischemic stroke. Yet, the underlying regulatory mechanism of miRNAs in cerebral ischemic stroke remains unclear. We investigated the role of miR-145 in cerebral ischemic stroke and its potential mechanism in a model using primary cultured astrocytes. We detected the expression levels of miR-145 and its target gene* AQP4* and assessed the role of miR-145 in cell death and apoptosis caused by oxygen-glucose deprivation (OGD). Bioinformatics analysis was used to explore the targets of miR-145. miR-145 expression levels were significantly decreased in primary astrocytes subjected to OGD. miR-145 overexpression promoted astrocyte health and inhibited OGD-induced apoptosis.* AQP4* was a direct target of miR-145, and miR-145 suppressed* AQP4* expression. Moreover, AQP4 enhanced astrocyte injury in ischemic stroke, and AQP4 knockdown diminished the miR-145-mediated protective effect on ischemic injury. Taken together, our results show that miR-145 plays an important role in protecting astrocytes from ischemic injury by downregulating* AQP4* expression. These findings may highlight a novel therapeutic target in cerebral ischemic stroke.

## 1. Introduction

 Cerebral ischemic stroke is one of the main diseases of the brain, with high incidence, mortality, and disability [1]. In China, ischemic stroke is the leading cause of death; over the past two decades, morbidity and mortality from stroke have dramatically increased in China [2]. Astrocytes play a dual role in ischemic stroke, either protecting neurons or exacerbating injury [3]. However, the rapid swelling of astrocytes in ischemic brain injury contributes to astrocyte dysfunction during stroke, and this rapid swelling is induced by aquaporins [4, 5]. Aquaporins are specialized water transport proteins that play an essential role in brain edema. Aquaporin 4 (AQP4) is highly expressed on astrocyte foot processes surrounding the capillaries [6]. Accumulating evidence has proven that AQP4 expression after cerebral ischemia is upregulated and that AQP4 knockdown reduces cytotoxic edema during stroke [7]. Moreover, it has been indicated that inhibiting AQP4 improved patient outcome and neurological function, reduced infarction volume, increased neuronal survival, and reduced apoptosis and the inflammatory response after cerebral ischemia, which was in accordance with brain edema reduction [8]. This role of AQP4 in brain edema indicates that astrocytes are the major cell type involved in cytotoxic edema during pathological processes such as stroke [9]. Therefore, inhibiting AQP4 channel function may be a potential target for ischemic stroke treatment. However, specific inhibitors and regulators of AQP4 channels have not been demonstrated. Hence, it is essential to explore potential AQP4 inhibitors and to uncover the regulatory mechanism of AQP4 expression. At the same time, such studies are conducive to elucidating the molecular mechanism of cerebral ischemic injury.

MicroRNAs (miRNAs) are highly conserved, endogenous ~22-nucleotide noncoding RNAs. miRNAs regulate target gene expression at posttranscriptional level by inhibiting transcription or by degrading mRNA [10]. miRNAs are involved in cancer, cardiovascular disease, and metabolic disorders [11–15]. Accumulating evidence indicates that miRNAs play a critical role in the pathophysiology of ischemic stroke by mediating angiogenesis, apoptosis, and oxidative stress [16]. For example, increased miR-181a exacerbated injury both in vitro and in a mouse stroke model by targeting G protein-coupled receptor 78 (GPR78). This suggests that miR-181a is a novel biomarker of cerebral ischemia [17]. miR-210 was upregulated in patients with ischemic stroke, and lentivirus-mediated miR-210 overexpression enhanced the microvessel density and the number of neural progenitor cells in ischemic mouse brain and improved neurobehavioral outcomes [18]. miR-455 significantly decreased primary neuronal cells subjected to oxygen-glucose deprivation (OGD) and mouse brain subjected to middle cerebral artery occlusion (MCAO). By downregulating tumor necrosis factor-associated factor 3 (TRAF3) protein expression, miR-455 played a vital role in protecting neuronal cells from death [19]. Large-scale microarray screening showed that miR-378 was downregulated in the peri-infarct region of MCAO mice, and miR-378 overexpression attenuated ischemic injury by negatively regulating the apoptosis executor caspase-3 [20]. miR-23a-3p was increased after reperfusion and played a protective role by attenuating oxidative injury in cerebral ischemia reperfusion [21]. The above studies demonstrate that miRNAs play an important role in ischemic stroke and that exploring more miRNAs to find effective treatment targets in ischemic stroke is essential. Gan et al. showed that circulatory miR-145 expression was significantly higher in patients with ischemic stroke [22]. Chen et al. demonstrated that miR-145 facilitated endothelial progenitor cell (EPC) proliferation and migration and arterial thrombosis recanalization in mice with cerebral infarction via the JNK signaling pathway [23]. These results indicate an important role of miR-145 in ischemic stroke; however, the precise mechanism of miR-145 in ischemic stroke has not been uncovered.

In the present study, we studied the role of miR-145 in astrocyte function by using the OGD model of cell ischemia in vitro. miR-145 protected astrocytes from OGD-induced injury. Furthermore, miR-145 regulated AQP4 expression in astrocytes and attenuated AQP4-induced astrocyte injury during OGD. Our study thereby highlights the fact that, by targeting AQP4, miR-145 might protect astrocytes from ischemia-induced injury.

## 2. Methods and Materials

### 2.1. Primary Astrocyte Culture

Primary astrocytes were prepared from 24 h postnatal neonatal Sprague-Dawley rats, and the protocol used was as described previously with minor changes [24]. The cells (1 × 10^6^ cells/ml) were plated onto 96-well plates and cultured in poly-L-lysine-coated 35-mm dishes with Dulbecco's modified Eagle's medium (DMEM) containing 10% bovine calf serum at 37°C in 5% CO_2_ in a humidified environment and allowed to grow to confluence.

### 2.2. Cell Transfection

The cells were trypsinized and plated onto 24-well plates, transfected on day 5 in vitro with miR-145 mimics or inhibitor or AQP4 small interfering RNA (siRNA) or controls using Lipofectamine 2000 from Invitrogen (Foster City, CA, USA) according to the manufacturer's instructions. The transfected cells were collected after 48 h for further experiments.

### 2.3. Immunofluorescence

The cells were fixed with 4% paraformaldehyde for 10 min at room temperature and then blocked with goat serum for 40 min at 37°C and incubated with glial fibrillary acidic protein (GFAP) (1 : 100; Abcam, Cambridge, MA, USA) overnight at 4°C. After washing with PBS three times, cells were incubated with secondary antibodies (1 : 100; Zhongshan Goldbridge Biotechnology, Beijing, China) for 1 h at 25°C. The nuclei were stained with diaminophenylindole (DAPI), and the cells were observed and photographed using a fluorescence microscope (IX81; Olympus, Tokyo, Japan).

### 2.4. Primary Astrocyte OGD Model

The primary astrocyte OGD model was established in accordance with previously described methods with minor modifications [25]. Briefly, the cells were transferred to glucose-free DMEM and cultivated in a humidified incubator with 95% N_2_ and 5% O_2_ at 37°C for 6 h. For reperfusion, the exposure medium was replaced with high-glucose DMEM, and the cells were incubated in a normoxic incubator for an additional 24 h. The cells cultures were assessed at the end of the treatment. Cells in the control group were cultured in plain DMEM and neuronal culture medium with ambient oxygen for 6–24 h (no OGD).

### 2.5. Quantitative Real-Time PCR (qRT-PCR)

At 48 h after transfection, total RNA, including miRNAs, was extracted using TRIzol (Invitrogen) according to the manufacturer's instructions. A NanoDrop ND1000 spectrophotometer (NanoDrop, Wilmington, DE, USA) was used to measure the RNA concentrations. First-strand complementary DNA was prepared from 2 *μ*g total RNA using a PrimeScript RT Reagent Kit (Takara, Dalian, China). SYBR Green PCR Master mix (Takara, Otsu, Japan) was used to determine* MIR145* and* AQP4* mRNA levels. All reactions were performed in triplicate. Relative miR-145 expression was normalized to the internal reference U6. Data were analyzed using the comparative threshold cycle value (2^−ΔΔCt^) method. The primers sequence used were as follows:

  AQP4: Forward 5′-CCCGCAGUUAUCAUGGGAATT-3′
     Reverse 5′-UUCCCAUGAUAACUGCGGGTT-3′
 
*β*-Actin: Forward 5′-TGGCACCCAGCACAATGAA-3′
      Reverse 5′-CTAAGTCATAGTCCGCCTAGAAGCA-3′
  U6: Forward 5′-ATTGGAACGATACAGAGAAGATT-3′
    Reverse 5′-GGAACGCTTCACGAATTTG-3′
  miR-145: Forward 5′-GUCCAGUUUUCCCAGGAAUCCCU-3′
      Reverse 5′-GGAUUCCUGGGAAAACUGGACUU-3′.

The sequence of synthesis was as follows:

  miR-145 mimics: Forward 5′-GUCCAGUUUUCCCAGGAAUCCCU-3′
         Reverse 5′-GGAUUCCUGGGAAAACUGGACUU-3′
  miR-145 inhibitor: 5′-AGGGAUUCCUGGGAAAACUGGAC-3′.

### 2.6. Apoptosis Assay

Apoptosis was analyzed using flow cytometry and annexin V/propidium iodide (PI) staining. After treatment, the cells were harvested and washed twice with phosphate-buffered saline (PBS), followed by the addition of annexin V-fluorescein isothiocyanate (FITC) and PI staining reagents (BD, Franklin Lakes, NJ, USA). The cells were resuspended with 200 *μ*l annexin V/PI premix buffer and then protected from light for 20 min at 4°C, following which 300 *μ*l binding buffer was added. Apoptotic cells were detected using annexin V-FITC/PI staining according to the protocol of an annexin V-FITC Apoptosis Detection Kit (BD).

### 2.7. Western Blotting

At 48 h after treatment, total proteins were extracted with radioimmunoprecipitation assay lysis buffer containing protease inhibitors and quantified using a Pierce Bicinchoninic Acid Protein Assay kit (IL, Rockford, USA). The proteins (30 *μ*g/lane) were electrophoretically separated using 10% sodium dodecyl sulfate polyacrylamide gel electrophoresis and transferred to polyvinylidene difluoride membranes. The membranes were incubated with 5% nonfat milk in Tris-buffered saline containing 0.1% Tween 20 (TBST) at room temperature for 1 h. The membranes were incubated with AQP4 antibody (1 : 1000; Abcam) and *β*-actin monoclonal antibody (1 : 5000; Abcam) at 4°C overnight. The membrane was incubated with secondary horseradish peroxidase-conjugated antibodies (1 : 2000; Abcam) for 2 h at room temperature. Immunoreactive proteins were visualized using an enhanced chemiluminescence-plus chemiluminescence reaction. The relative AQP4 content is represented as the grayscale ratio of AQP4/*β*-actin, and the grayscale was analyzed using Quantity One software (Bio-Rad, Hercules, CA, USA).

### 2.8. Enzyme-Linked Immunosorbent Assay (ELISA)

The levels of lactate dehydrogenase (LDH) were determined using an ELISA kit according to the manufacturer's instructions (Roche, Basle, Switzerland). The LDH concentrations were calculated according to the absorbance of the samples and the standard curve.

### 2.9. Cell Health Assay

According to the manufacturer's protocol (CST, Danvers, MA, USA), calcein acetoxymethyl ester (calcein-AM)/PI staining was used to measure cell health. Briefly, 1 × 10^5^ cells were plated in 96-well plates in warm culture medium and cultured in an incubator overnight to allow the cells to attach to the plate. Then, the medium was removed and the cells were washed once with 1 × PBS, 100 *μ*l/well labeling solution was added to the plate, and the cells were incubated at room temperature for 30–60 min away from light. Then, the cells were analyzed on a plate reader set at 490/520 nm and 535/620 nm excitation/emission for live cells and dead cells, respectively.

### 2.10. Luciferase Reporter Assay

Fragment of the 3′UTR and mutant 3′UTR of AQP4 was amplified and then cloned into pGL3 vector which contains the firefly luciferase reporter gene (Promega, Madison, WI, USA). For the luciferase reporter assay, rat astrocytes were cotransfected with 200 ng firefly luciferase constructs, 4 ng pRL-TK* Renilla* luciferase plasmid, and 50 nM miR-145-5p mimics.* Renilla* luciferase activity was measured using a dual-luciferase reporter assay (Promega) 48 hours after transfection. The results were expressed as relative luciferase activity (firefly luciferase/*Renilla* luciferase).

### 2.11. Statistical Analysis

All data are expressed as the mean ± SEM from three independent experiments (each in duplicate). The Student* t*-test and one-way analysis of variance, followed by the post hoc Scheffe test, were used for statistical analysis using SPSS 18.0 software (SPSS, Chicago, IL, USA). *p* < 0.05 was considered statistically significant.

## 3. Results

### 3.1. miR-145 Overexpression Suppressed OGD-Induced Astrocyte Injury

**Figure 1:**
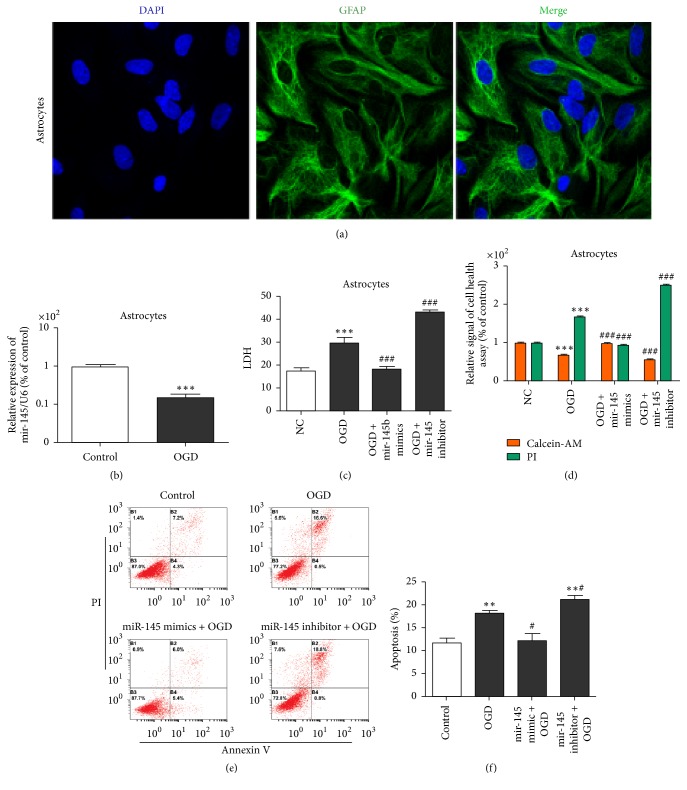
miR-145 protected astrocytes from OGD-induced injury. (a) GFAP/DAPI staining of primary astrocytes (×200 magnification). (b) qRT-PCR detection of miR-145 expression in OGD primary cultured astrocytes or NC; U6 was used as the internal control (^*∗∗∗*^*p* < 0.001 versus control). (c) ELISA of LDH levels in astrocyte culture supernatant (^###^*p* < 0.001 versus OGD; ^*∗∗∗*^*p* < 0.001 versus control). (d) Calcein-AM/PI staining cell health assay. PI-positive cells were dead cells; calcein-AM-positive cells were healthy cells (^###^*p* < 0.001 versus OGD; ^*∗∗∗*^*p* < 0.001 versus control). (e) Flow cytometry assay of astrocyte apoptosis assay. Cells in the B2 and B4 quadrants represent apoptotic cells. (f) The percentage of apoptotic cells in each group. ^*∗*^*p* < 0.05 versus NC group; ^#^*p* < 0.05 versus OGD group; ^*∗∗*^*p* < 0.01 versus control.

To investigate the biological role of miR-145 in astrocytes after OGD, we analyzed astrocytes transfected with miR-145 mimics or inhibitor. We first identified the extracted primary astrocytes using GFAP immunofluorescence staining (Figure 1(a)). The cell experiments demonstrated that miR-145 expression was obviously decreased in OGD astrocytes as compared with the controls (Figure 1(b)). Next, we examined the cell supernatant LDH levels, cell health, and apoptosis of the treated primary cultured astrocytes. Cell supernatant LDH levels (Figure 1(c)), cell health (Figure 1(d)), and apoptosis (Figures 1(e) and 1(f)) were increased after 6 h OGD. miR-145 upregulation inhibited OGD-induced LDH release, cell health, and apoptosis, whereas miR-145 downregulation had the opposite effect (Figures 1(c)–1(f)). The results suggest that upregulating miR-145 regulates astrocyte survival positively after ischemic brain injury.

### 3.2. AQP4 Was a Functional Target of miR-145

**Figure 2:**
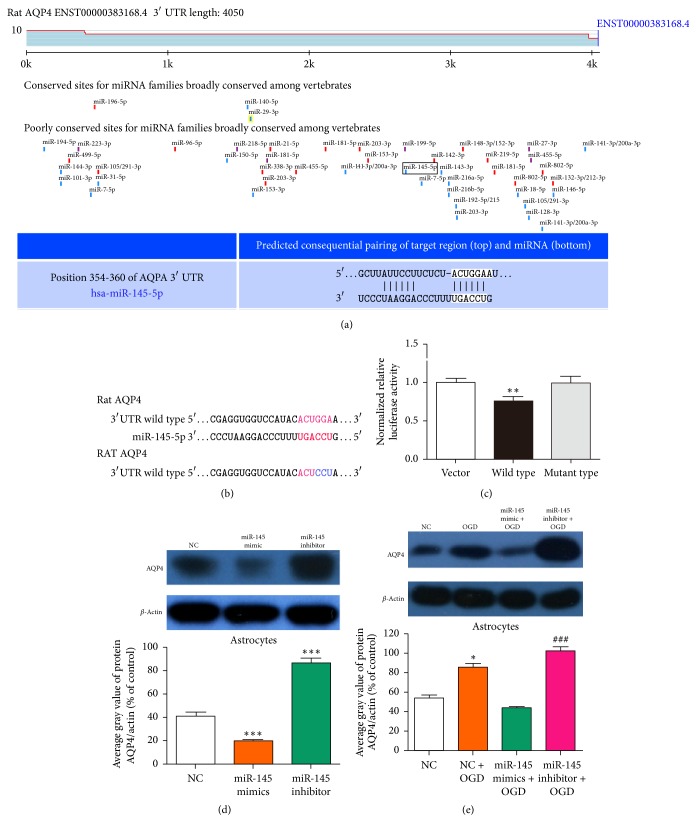
*AQP4* was the target gene of miR-145. (a) Sequence alignment indicating nucleotide complementarity between miR-145 and the 3′ UTR of AQP4. (b) Schematic representation of miR-145-5p recognition site in AQP4 3′ untranslated region (UTR) and AQP4 mut-3′ UTR. (c) Bar graph represented normalized relative luciferase activity after cotransfection with miR-145-5p mimics (50 nM) in blank vector, wild type, and mutant type. ^*∗∗*^*p* < 0.01 versus vector, data are mean ± s.d., three independent experiments; ^*∗∗*^*p* < 0.01 versus vector. (d) Effect of miR-145 on AQP4 protein levels in primary astrocytes; *β*-actin was used as the internal control (^*∗∗∗*^*p* < 0.001 versus NC). (e) Effect of miR-145 on AQP4 protein levels in primary astrocytes with or without OGD; *β*-actin was used as the internal control. ^*∗*^*p* < 0.05 versus NC group; ^#^*p* < 0.05 versus miR-145 mimics + OGD group; ^###^*p* < 0.001 versus OGD miR-145 mimics + OGD group.

The miRBase (http://www.mirbase.org/) and TargetScan 5.1 (http://www.targetscan.org/) miRNA databases were used to define the target genes of miR-145 in regulating ischemic stroke. The results indicated that AQP4 was a target of miR-145 (Figure 2(a)). To confirm whether AQP4 was regulated by miR-145-5p, we cloned AQP4 mRNA 3′UTR fragment and mutant 3′UTR fragment containing the putative miR-145-5p binding sites upstream of the luciferase coding sequence and performed cotransfection of the luciferase reporter and miR-145-5p mimics in rat astrocytes (Figure 2(b)). Luciferase activity level was reduced in the cells cotransfected with miR-145-5p mimics and AQP4 mRNA 3′UTR fragment, but not the miR-145-5p mimic and the mutant 3′UTR fragment group (Figure 2(c), ^*∗∗*^*p* < 0.01). These results suggest that AQP4 is a direct target of miR-145-5p. Therefore, we hypothesized that miR-145 may exert its function in ischemic stroke by targeting* AQP4*. To prove this hypothesis, we assessed AQP4 protein levels in astrocytes transfected with miR-145 mimics or inhibitor or negative control (NC). miR-145 upregulation significantly reduced AQP4 protein levels in the astrocytes, and miR-145 inhibitor significantly increased them (Figure 2(d)). Furthermore, AQP4 expression was increased in OGD astrocytes (Figure 2(e)). Above all, miR-145 upregulation inhibited OGD-induced AQP4 protein expression in the astrocytes, whereas miR-145 downregulation promoted it (Figure 2(e)). These results indicate that miR-145 targets* AQP4* and negatively regulates AQP4 expression by binding to the* AQP4* 3′ untranslated region (3′ UTR).

### 3.3. AQP4 Knockdown Suppressed OGD-Induced Astrocyte Injury

**Figure 3:**
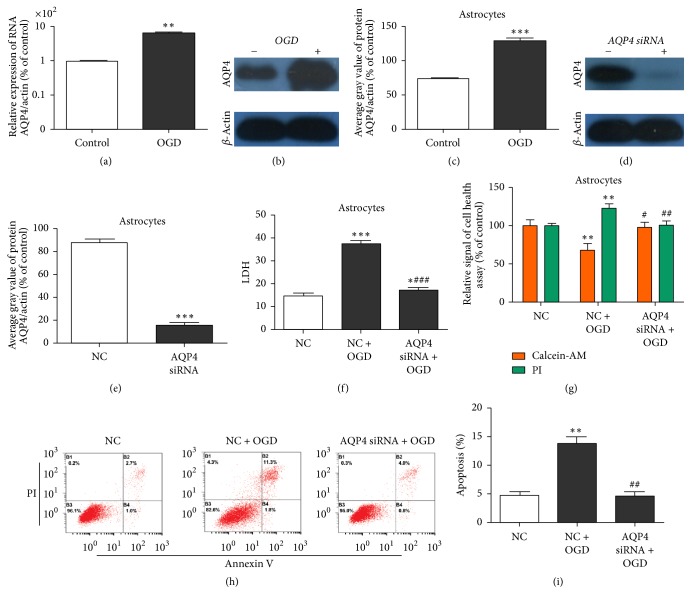
AQP4 knockdown inhibited OGD-induced primary astrocyte injury. (a) Real-time PCR detection of* AQP4* mRNA levels in OGD astrocytes or NC; *β*-actin was used as the internal control (^*∗∗*^*p* < 0.01 versus control). (b, c) Western blots of AQP4 protein levels in OGD astrocytes or NC; *β*-actin was used as the internal control (^*∗∗∗*^*p* < 0.001 versus control). (d, e) Western blotting identification of AQP4 siRNA efficiency; *β*-actin was used as the internal control (^*∗∗∗*^*p* < 0.001 versus NC). (f) ELISA of LDH levels in astrocyte culture supernatant (^*∗∗∗*^*p* < 0.001 versus NC). (g) Calcein-AM/PI staining cell health assay. PI-positive cells are dead cells; calcein-AM-positive cells are healthy cells (^*∗*^*p* < 0.05, ^*∗∗*^*p* < 0.01 versus NC; ^#^*p* < 0.05; ^##^*p* < 0.01 versus NC + OGD; ^###^*p* < 0.001 versus OGD). (h, i) Flow cytometry assay of astrocyte apoptosis. Cells in the B2 and B4 quadrants are apoptotic cells (^*∗∗*^*p* < 0.01 versus NC group; ^##^*p* < 0.01 versus NC + OGD group). ^*∗*^*p* < 0.05 versus NC group; ^#^*p* < 0.05 versus NC + OGD group.

The function of AQP4 in OGD astrocytes was investigated to confirm whether miR-145 protects astrocytes from OGD-induced injury by regulating AQP4 expression. We examined AQP4 expression in OGD astrocytes and found that AQP4 protein and mRNA levels were obviously upregulated in those cells as compared with the control group (Figures 3(a)–3(c)). Next, AQP4 was knocked down in the astrocytes; AQP4 siRNA significantly downregulated AQP4 levels (Figures 3(d) and 3(e)). To study the role of AQP4 in OGD-induced injury, we examined the cell supernatant LDH levels, cell health, and apoptosis of the primary cultured astrocytes. AQP4 knockdown had the same effects as compared with miR-145 in OGD astrocytes, significantly decreasing the levels of released LDH, cell health, and apoptosis (Figures 3(f)–3(i)).

### 3.4. AQP4 Downregulation Decreased miR-145-Mediated Astrocyte Protection

**Figure 4:**
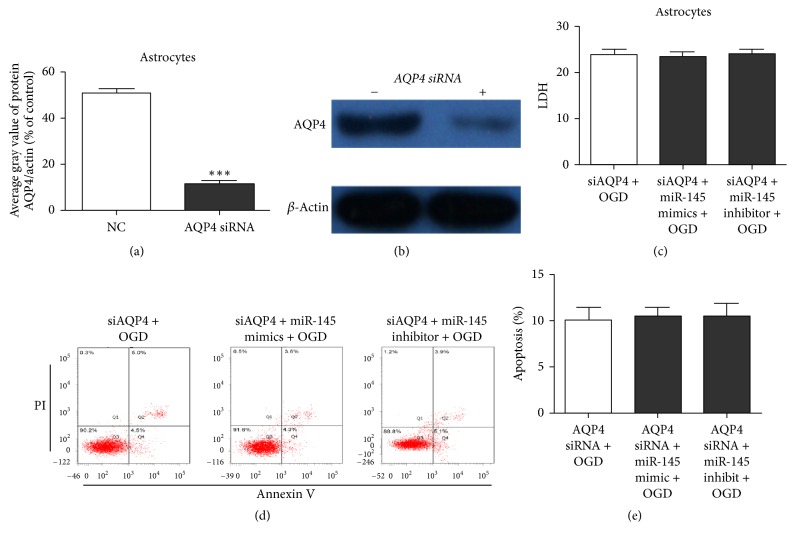
AQP4 knockdown diminished the protective effect of miR-145 in astrocytes. (a, b) Western blotting identification of AQP4 siRNA efficiency; *β*-actin was used as the internal control. (c) ELISA of LDH levels in astrocyte culture supernatant. (d, e) Flow cytometry assay of astrocyte apoptosis. Cells in the B2 and B4 quadrants are apoptotic cells.

We transfected OGD astrocytes with AQP4 siRNA plus miR-145 mimics or inhibitor or with AQP4 siRNA alone to determine the relationship between miR-145 and AQP4. The interference efficiency of AQP4 siRNA was confirmed by western blotting and represented as a histogram based on the gray value (Figures 4(a) and 4(b)). As reported above, miR-145 prevented LDH release and apoptosis in OGD astrocytes, but the addition of AQP4 siRNA decreased the protective effect of the miR-145 mimics. The miR-145 inhibitor significantly promoted astrocyte injury; however, AQP4 siRNA also diminished the effect (Figures 4(c)–4(e)). These findings prove that AQP4 mediates the protective role of miR-145 in cerebral ischemia.

## 4. Discussion

Cerebral ischemia, frequently induced through various pathological pathways, results in irreversible neuronal injury in the ischemic region. Increasing evidence indicates that astrocytes play an important role in stroke [3]. Here, we investigated the effects of miR-145 on OGD-induced astrocyte injury. Our results provide novel insights into the molecular mechanism of miR-145-mediated astrocyte protection in stroke at miRNA level. Further, our results indicate the possibility of miR-145 as an endogenous miRNA agonist for stroke.

Accumulating evidence has demonstrated that miRNAs play vital roles in cerebral ischemia. miRNAs participate in various biological events, including the inflammatory response, edema, and apoptosis, which are related to cerebral damage [26, 27]. Exploration of the miRNAs associated with ischemic stroke could provide new insights into new therapeutic approaches. For example, miR-24 inhibitor prevented neuron apoptosis in ischemic stroke by regulating Bcl-xL, caspase-3, and heat shock protein 70 (HSP70) [28]. miR-181b knockdown alleviated ischemic injury by upregulating HSPA5 and ubiquitin C-terminal hydrolase L1 (UCHL1) expression [29]. Similarly, miR-30a inhibition mediated neuroprotection from ischemic injury by upregulating HSPA5 levels in vitro and in vivo [30]. miR-424 treatment exacerbated H_2_O_2_-induced LDH leakage and promoted manganese superoxide dismutase (SOD) activity in neurons, and inhibiting nuclear factor erythroid 2-related factor (NRF-2) and SOD alleviated its protective role against oxidative stress [31]. Gan et al. showed that the expression of circulatory miR-145 was significantly higher in patients with ischemic stroke [22]. Chen et al. demonstrated that, in mice with cerebral infarction, miR-145 facilitated EPC proliferation and migration and the recanalization of arterial thrombosis via the JNK signaling pathway [23]. These results indicate the important role of miR-145 in ischemic stroke. Consistent with this, we demonstrate that miR-145 levels were decreased in astrocytes after OGD. Importantly, miR-145 overexpression in the astrocytes significantly attenuated OGD-induced injury, suggesting that miR-145 may be a promising therapeutic target for cerebral ischemic stroke.

AQP4 is the major expressed water transport channel in the central nervous system and is predominantly anchored in astrocyte foot processes surrounding the capillaries. It is crucial for the formation and resolution of brain edema [32]. Increasing evidence indicates that AQP4 plays an important role in enhancing brain ischemia injury. Studies on AQP4 expression after cerebral ischemia have mainly been performed in animal or cell models. Lu et al. demonstrated that AQP4 mRNA and protein are upregulated at 30 min after permanent MCAO [33]. A continuous and dynamic observation study on a MCAO and reperfusion model reported that AQP4 expression was significantly upregulated on astrocyte end-feet with two peaks: 1 h and 48 h [34]. In an astrocyte culture, Nito et al. showed that OGD injury significantly decreased AQP4 levels, but reoxygenation gradually restored the AQP4 levels, with significant upregulation after 16 h [35]. Moreover, it has been indicated that AQP4 knockout improved outcome and neurological function, reduced infarction volume, increased neuronal survival, and blocked apoptosis and inflammatory response after cerebral ischemia, which is consistent with brain edema reduction [7]. In vivo and in vitro experiments on ischemic stroke have reported that specific signal transduction pathways regulate AQP4 expression. The mitogen-activated protein kinase (MAPK) pathways play an important role in AQP4 upregulation in ischemic stroke [36–38]. Moreover, several miRNAs, such as miR-29b and miR-130a, regulate AQP4 expression in cerebral ischemia [39, 40]. Here, we used online software, miRBase and TargetScan, and found that miR-145 regulates AQP4 expression at posttranscriptional level by binding to the 3′ UTR of* AQP4* mRNA. We further proved that AQP4 downregulation rescues astrocyte ischemic injury. AQP4 silencing attenuated the protective effect of the miR-145 mimics on astrocytes. Therefore, our study provides strong evidence that miR-145 prevents astrocyte injury by inhibiting AQP4.

In conclusion, our results reveal that miR-145 protects astrocytes from injury after OGD by inhibiting AQP4 expression. Therefore, miR-145 functions as a novel regulator in stroke and may be developed into a protective medicine for treating stroke. Considering this, further investigation of the elucidation of the miRNA mechanisms involved in the potential pathogenesis of cerebral ischemia is warranted.
